# Resistance to SMO Inhibitors in Advanced Basal Cell Carcinoma: A Case Highlighting the Role of Molecular Tumor Profiling

**DOI:** 10.3390/ijms27010068

**Published:** 2025-12-21

**Authors:** Federica Papaccio, Ramona Marrapodi, Laura Eibenschutz, Andrea D’Arino, Silvia Caputo, Alberto Marini, Simona Scano, Arianna Presaghi, Carlo Cota, Elisa Melucci, Stefano Scalera, Emilia Migliano, Marcello Maugeri-Saccà, Pasquale Frascione, Barbara Bellei

**Affiliations:** 1 Laboratory of Cutaneous Physiopathology and Integrated Center of Metabolomics Research, San Gallicano Dermatological Institute, IRCCS, Via Elio Chianesi 53, 00144 Rome, Italy; 2 Oncologic and Preventative Dermatology, San Gallicano Dermatological Institute, IRCCS, Via Elio Chianesi 53, 00144 Rome, Italy; 3 Department of Plastic and Regenerative Surgery, San Gallicano Dermatological Institute, IRCCS, Via Elio Chianesi 53, 00144 Rome, Italy; arianna.presaghi@ifo.it (A.P.);; 4 Genetic Research, Molecular Biology and Dermatopathology Unit, San Gallicano Dermatological Institute, IRCCS, Via Elio Chianesi 53, 00144 Rome, Italy; 5 Department of Pathology, Regina Elena National Cancer Institute, IRCCS, Via Elio Chianesi 53, 00144 Rome, Italy; 6 Biostatistics, Bioinformatics and Clinical Trial Center, Regina Elena National Cancer Institute, IRCCS, Via Elio Chianesi 53, 00144 Rome, Italy

**Keywords:** basal cell carcinoma, hedgehog pathway, drug resistance, targeted therapies

## Abstract

Basal cell carcinoma (BCC) is the most common skin cancer, predominantly affecting sun-exposed areas. It typically grows slowly and rarely metastasizes, though untreated cases can cause significant tissue destruction and morbidity. Its pathogenesis primarily involves dysregulation of the Hedgehog (HH) signaling pathway, mainly through mutations in *PTCH1* or *SMO* genes, leading to chronic activation of downstream GLI transcription factors. Accordingly, current targeted therapies for locally advanced, unresectable, or metastatic BCC focus on SMO inhibition, using orally administered drugs such as vismodegib and sonidegib. Although these therapies have shown success, many patients develop resistance, with about 50% harboring mutated SMO. In numerous cases, genetic determinants (sometimes pre-existing) of resistance remain unidentified, complicating patient management. Here, we report a case of a 58-year-old female with advanced BCC who initially exhibited a favorable response to sonidegib but developed resistance after approximately one year. This resistance was not attributable to the acquired mutations in *SMO* but rather to intra-tumor heterogeneity and additional mutations in critical driver genes, including *TP53*, *APC*, *FGFR1* and *NOTCH1*, which likely enable HH pathway inhibition. To our knowledge, this is the first report documenting a sonidegib resistance mechanism in BCC that is independent of HH pathway mutations. This case highlights the complexity of resistance mechanisms to HH inhibitors and underscores the critical need for comprehensive molecular tumor profiling prior to initiating targeted therapy.

## 1. Introduction

Basal cell carcinoma (BCC) is the most common form of skin cancer worldwide [1,2]. In certain cases, especially locally advanced or metastatic BCC, the disease can cause significant morbidity and treatment challenges [3]. BCC development is strictly linked to abnormal activation of the Hedgehog (HH) signaling pathway, often due to loss-of-function mutations in the *Patched 1* (*PTCH1*) gene or gain-of-function mutations in the *Smoothened* (*SMO*) receptor [4]. Less commonly, mutations occur downstream in *suppressor of fused* (*SUFU*) or *glioma-associated transcription factors* (*GLI*) [5]. These changes lead to chronic sustained activation of HH signaling, supporting uncontrolled cell growth. Cyclopamine-competitive antagonists of SMO, like vismodegib and sonidegib, are approved for BCC patients with locally advanced or metastatic BCC unsuitable for surgery or radiation, helping control the disease and reducing tumor size [6]. These molecules alone or in combination with immunotherapy are also undergoing clinical trials for several solid tumors (clinicaltrials.gov). Despite clinical success, resistance often develops. It frequently stems from *SMO* mutations that prevent drug binding or from the copy number of *SUFU*, *tumor protein 53* (*TP53*), *GLI2* and *PTCH1* [7]. Understanding these mechanisms is essential for developing new treatments that overcome resistance and improve patient outcomes. This case report describes a patient with advanced BCC who developed sonidegib resistance in a complex molecular context. It highlights management challenges and the value of initial molecular profiling for guiding effective therapies.

## 2. Case Report

**Figure 1:**
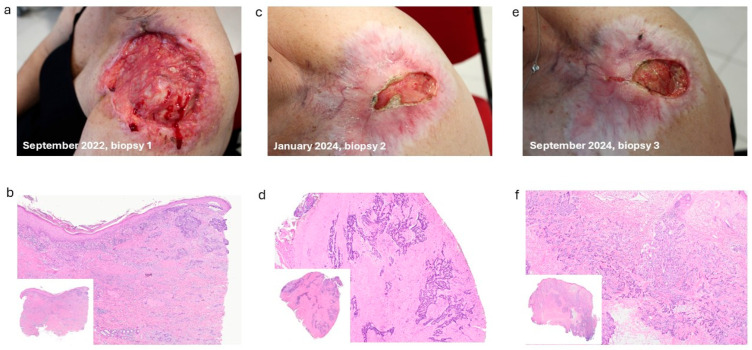
Clinical and histological findings. (**a**) Clinical photograph at initial diagnosis (September 2022) showing an ulcerated and bleeding lesion involving the entire anterior deltoid region of a 58-year-old female. (**c**) Local relapse was observed approximately one year after (January 2024) sonidegib treatment. (**e**) Lesion progression after an additional nine months (September 2024). (**b**) Histological findings from the initial biopsy. (**d**) Histopathological findings corresponding to panel (**c**). (**f**) Histopathological findings corresponding to panel (**e**). Original magnifications 4× and 10× respectively.

A 58-year-old female patient first presented in September 2022 with a large, ulcerated, and bleeding lesion over the left acromion, measuring approximately 8 × 7 cm and involving the entire anterior deltoid region (Figure 1a). The lesion had been neglected for several months due to personal family issues, and no previous treatment had been undertaken. A skin biopsy confirmed the diagnosis of micronodular infiltrative BCC (Figure 1b).

A concurrent lymph node ultrasound revealed a suspicious heterogeneous mass in the left supraclavicular region, measuring 2.6 × 4.2 cm, with polycyclic and ill-defined margins. The lesion was located between the skin and clavicle, exhibiting rich vascularization on color Doppler imaging. Systemic treatment with sonidegib 200 mg daily was initiated, resulting in a favorable clinical response as early as the second treatment cycle, characterized by progressive reduction in both lesion size and depth. Follow-up ultrasound in March 2023 confirmed the improvement in the supraclavicular lymph node involvement. The clinical response persisted until October 2023, when a persistent ulcerated area developed in the lateral portion of the lesion, over the deltoid region. Due to progressive worsening, additional biopsies were performed in January 2024 (Figure 1c), confirming persistent micronodular infiltrative BCC (Figure 1d). In July 2024, a total-body computed tomography (CT) scan revealed multiple bilateral nodular and pseudonodular pulmonary opacities, some with air bronchograms, the largest measuring approximately 15 mm in the dorsal segment of the left lower lobe, with indeterminate characteristics. A Positron Emission Tomography combined with computed tomography (PET/CT) scan and infectious disease consultation was obtained. In August 2024, PET/CT demonstrated metabolically active pathological tissue involving the left shoulder with extensive infiltration of muscular and skeletal tissues, as well as pulmonary and lymph node metastases. Multiple nodular lesions were present in both lungs, including the dorsal segment of the left lower lobe (SUVmax 9.2), the apical segment of the right upper lobe (SUVmax 8.3), and the anterior lingular segment of the left lung in a parahilar location (SUVmax 8.4). As the clinical course progressed, the ulcer continued to worsen (Figure 1e); consequently, the patient underwent an additional biopsy in September 2024, which documented micronodular infiltrative BCC (Figure 1f). The patient was subsequently screened for treatment escalation with cemiplimab, an immune checkpoint inhibitor that blocks the interaction of programmed cell death-1 (anti-PD-1) with its ligand [8]. The patient is currently undergoing cemiplimab treatment at another institution, with a good response in the pulmonary lesions and a partial response in the cutaneous disease. Next to clinical evaluation, we analyzed genomic DNA extracted from formalin-fixed, paraffin-embedded biopsies to distinguish intrinsic pre-existing resistance from acquired post-treatment resistance. The emergence after an intermediate period (one year) could result from either mechanism. Targeted next-generation sequencing (NGS) was performed on the Illumina platform using a custom-designed gene panel (ID 3521071, Agilent) covering ten HH pathway-related genes (*SHH*, *DHH*, *IHH*, *GLI1*, *GLI2*, *GLI3*, *PTCH1*, *PTCH2*, *SMO*, *SUFU*), alongside a commercially available multigene NGS panel targeting 50 genes Thermofischer Scientific (CHPV2). The analysis of material corresponding to the disease onset evidenced mutations in *APC*, *FGFR1*, *TP53*, and *NOTCH1* genes (Table 1). 

**Figure 2:**
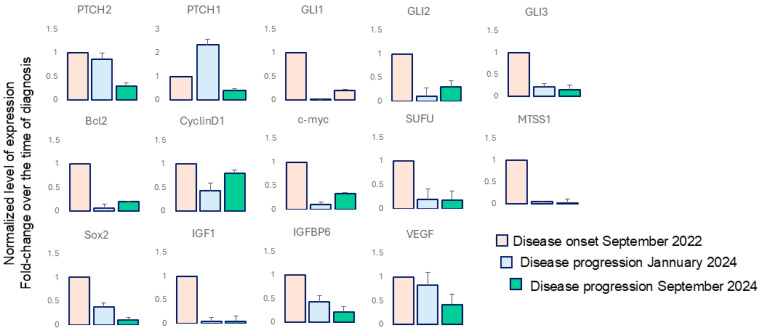
Expression profile of HH target genes. Comparative analysis of mRNA of a panel of HH target gene. Data evidenced the persistence of HH signaling repression during the therapy with sonidegib. Samples were analyzed in triplicate by RT-PCR (see Supplementary Material section).

At the relapse, the patterns of mutations were confirmed, but the additional p.Leu412Phe_c.1234C > T missense variant in the *SMO* sequence, with a variant allele frequency (VAF) of 14%, was detected. The detected *SMO* mutation has not been previously reported as conferring therapy resistance. This finding aligns with the observation that sonidegib effectively eliminated cells with activated HH signaling, as not *SMO* mutation was evident at disease progression. Accordingly, the expression of target genes, assessed by RT-PCR, including transcription factors (*SOX2*), apoptosis regulators (*BCL2*), and cell cycle regulators (*MYC*, *CCDN1*), IGF signaling regulators (*IGF* and *IGFBP6*), critical promoter of vasculogenesis (VEGF) as well as HH pathway feedback genes indicative of pathway activation, such as *PTCH2*, and *GLI1*, were downregulated during treatment, even in presence of clinical disease relapse (Figure 2). Methods and oligonucleotide sequences are reported in Supplemental Material, Table S1.

The modest recovery of expression observed for some mRNAs in the final biopsy may be explained by the interconnected regulation between HH and NOTCH signaling, as Notch signaling helps maintain HH responsiveness. Overall, data indicate a heterogeneous disease composed of multiple tumor clones, some harboring genomic alterations implicated in sonidegib resistance.

## 3. Discussion

The emergence of resistance to SMO inhibitors in BCC represents a well-recognized clinical challenge, often linked to secondary mutations that restore HH pathway activity or activate compensatory signaling routes. Previous reports identified genetic alteration of SMO as the prevalent mechanism by which tumors evade SMO inhibitor therapy. Overall, two mechanisms of acquired SMO resistance in BCC have been delineate: 50% of inhibitor-resistant cases harbored mutations in the drug-binding site or sequence variants disrupting autoinhibition, thereby causing constitutive SMO activity [9]. Non-SMO resistance involved downstream factors such as reduced SUFU copy numbers and elevated GLI2 amplification. In the presented case, no mutational events involving HH signaling components were detected at initial diagnosis. However, during treatment, a mutation in *SMO*, previously reported in aggressive BCC forms [10], occurred. However, this evolution did not significantly affect the efficacy of sonidegib treatment, as cells harboring this variant were effectively eliminated by the therapy. This finding confirms the efficacy of HH pathway inhibitors against tumor cells carrying mutated components of the signaling cascade. Here, tumor heterogeneity rather than the acquisition of secondary activating alterations represents a different mechanism of resistance to SMO inhibition. Nonetheless, concurrent alteration of the coding sequence of the *NOTCH1* gene persisted across all time points with a high VAF, suggesting a possible mechanism for the therapeutic escape. Notably, NOTCH signaling activation emerges as a common feature of non-target mutation resistance in cancer, suggesting its central role in promoting cancer cell persistence [11]. Several Notch pathway inhibitors are currently under phase II clinical investigation for hematologic and solid malignancies (clinicaltrials.gov). Notably, the combination of RO4929097, a γ-secretase inhibitor that blocks NOTCH signaling, with vismodegib has been proposed for advanced sarcoma [12] and breast cancer (NCT01071564). Thus, targeting Notch signaling might represent a complementary therapeutic strategy in selected BCC cases. Mutations in Notch family genes in BCC have been reported with different frequencies: Bonilla and collaborators disclosed *NOTCH1* and *NOTCH2* mutations were observed in 26% and 29% of BCCs, respectively [13], whereas another study indicates a frequency of about 43.8% for *NOTCH1* [14]. Other potential targets emerged from NGS analysis during tumor evolution, since the frequency for *APC*, *FGFR1* and *TP53* variants increased over time. Mutated *APC* promotes WNT signaling activation favoring tumor regrowth during HH inhibitors treatment following an early response [15]. Future experimental studies will more thoroughly investigate the specific role of this mutational pattern in sonidegib resistance in BCC.

This case illustrates the dynamic nature of cutaneous BCC, involving secondary HH pathway alterations alongside intra-tumor heterogeneity as a distinct resistance mechanism. These findings underscore the complexity of resistance in BCC and highlight the need for comprehensive molecular profiling. Such profiling guides effective treatment strategies, as resistance may emerge from diverse clones with genetic changes beyond SMO mutations. The interpretation is consistent with current evidence showing that while some *SMO* mutations confer resistance to inhibitors like sonidegib, not all mutations do, and tumor heterogeneity plays a crucial role in therapeutic outcomes. This case highlights the intricate resistance mechanisms to HH pathway inhibitors and stresses the necessity of detailed molecular profiling of tumors before targeted treatment. Future therapeutic approaches may require co-targeting or combining therapies to overcome resistance.

## Figures and Tables

**Table 1:** Detailed description of mutations found in the three biopsies.

Locus	Type	Genes	Location	September 2022	VAF	January 2024	VAF	September 2024	VAF
chr5:112175245	SNV	APC	NM_000038.6	p.Pro1319Ser_c.3955C>T	*5.90%*	p.Pro1319Ser_c.3955C>T	*27.30%*	p.Pro1319Ser_c.3955C>T	*30.20%*
chr7:128846398	SNV	SMO	NM_005631.5	N.D.		p.Leu412Phe_c.1234C>T	*14.20%*	N.D.	
chr8:38285875	SNV	FGFR1	NM_001174067.1	p.Pro179Leu_c.536C>T	*9.60%*	p.Pro179Leu_c.536C>T	*21.50%*	p.Pro179Leu_c.536C>T	*23.70%*
chr9:139390794	SNV	NOTCH1	NM_017617.5	p.Thr2466Met_c.7397C>T	*43.20%*	p.Thr2466Met_c.7397C>T	*58.70%*	p.Thr2466Met_c.7397C>T	*53.60%*
chr17:7577094	SNV	TP53	NM_000546.6	p.Arg282Trp_c.844C>T	*6.20%*	p.Arg282Trp_c.844C>T	*24.90%*	p.Arg282Trp_c.844C>T	*25.90%*
chr17:7577111	SNV	TP53	NM_000546.6	p.Ala276Val_c.827C>T	*6.30%*	p.Ala276Val_c.827C>T	*24.60%*	p.Ala276Val_c.827C>T	*25.10%*
chr17:7578368	MNV	TP53	NM_000546.6	p.Pro177Leu_c.530_531delCCInsTT	*5.90%*	p.Pro177Leu_c.530_531delCCInsTT	*31%*	p.Pro177Leu_c.530_531delCCInsTT	*27.50%*

## Data Availability

The data that support the findings of this study are available on request from the corresponding author. The data are not publicly available due to privacy or ethical restrictions.

## Supplementary Materials

The following supporting information can be downloaded at: https://www.mdpi.com/article/10.3390/ijms27010068/s1. Reference [16] is cited in the supplementary materials.

## References

[1] Rubin A.I., Chen E.H., Ratner D. (2005). Basal-Cell Carcinoma. N. Engl. J. Med..

[2] Verkouteren J.A.C., Ramdas K.H.R., Wakkee M., Nijsten T. (2017). Epidemiology of Basal Cell Carcinoma: Scholarly Review. Br. J. Dermatol..

[3] Puig S., Berrocal A. (2015). Management of High-Risk and Advanced Basal Cell Carcinoma. Clin. Transl. Oncol..

[4] Bakshi A., Chaudhary S.C., Rana M., Elmets C.A., Athar M. (2017). Basal Cell Carcinoma Pathogenesis and Therapy Involving Hedgehog Signaling and Beyond. Mol. Carcinog..

[5] Farage M.A., Miller K.W., Berardesca E., Maibach H.I. (2009). Clinical Implications of Aging Skin: Cutaneous Disorders in the Elderly. Am. J. Clin. Dermatol..

[6] Markota Cagalj A., Glibo M., Karin-Kujundzic V., Serman A., Vranic S., Serman L., Skara Abramovic L., Bukvic Mokos Z. (2025). Hedgehog Signalling Pathway Inhibitors in the Treatment of Basal Cell Carcinoma: An Updated Review. J. Drug Target..

[7] Sharpe H.J., Pau G., Dijkgraaf G.J., Basset-Seguin N., Modrusan Z., Januario T., Vsui V., Durham A.B., Dlugosz A.A., Haverty P.M. (2015). Genomic analysis of smoothened inhibitor resistance in basal cell carcinoma. Cancer Cell.

[8] Untaaveesup S., Srichana P., Techataweewan G., Pongphaew C., Dendumrongsup W., Ponvilawan B., Nampipat N., Limwongse C. (2025). Prevalence of Genetic Alterations in Basal Cell Carcinoma Patients Resistant to Hedgehog Pathway Inhibitors: A Systematic Review. Ann. Med..

[9] Atwood S.X., Sarin K.Y., Whitson R.J., Li J.R., Kim G., Rezaee M., Ally M.S., Kim J., Yao C., Chang A.L.S. (2015). Smoothened n Variants Explain the Majority of Drug Resistance in Basal Cell Carcinoma. Cancer Cell.

[10] Huang X., Chen W., Wang Y., Shytikov D., Wang Y., Zhu W., Chen R., He Y., Yang Y., Guo W. (2025). Canonical and noncanonical NOTCH signaling in the nongenetic resistance of cancer: Distinct and concerted control. Front. Med..

[11] Gounder M.M., Rosenbaum E., Wu N., Dickson M.A., Sheikh T.N., D’Angelo S.P., Chi P., Keohan M.L., Erinjeri J.P., Antonescu C.R. (2022). A Phase Ib/II Randomized Study of RO4929097, a Gamma-Secretase or Notch Inhibitor with or without Vismodegib, a Hedgehog Inhibitor, in Advanced Sarcoma. Clin. Cancer Res..

[12] Smith D.C., Chugh R., Patnaik A., Papadopoulos K.P., Wang M., Kapoun A.M., Xu L., Dupont J., Stagg R.J., Tolcher A. (2019). A Phase 1 Dose Escalation and Expansion Study of Tarextumab (OMP-59R5) in Patients with Solid Tumors. Investig. New Drugs.

[13] Bonilla X., Parmentier L., King B., Bezrukov F., Kaya G., Zoete V., Seplyarskiy V.B., Sharpe H.J., McKee T., Letourneau A. (2016). Genomic Analysis Identifies New Drivers and Progression Pathways in Skin Basal Cell Carcinoma. Nat. Genet..

[14] Di Nardo L., Pellegrini C., Di Stefani A., Ricci F., Fossati B., Del Regno L., Carbone C., Piro G., Corbo V., Delfino P. (2021). Molecular Alterations in Basal Cell Carcinoma Subtypes. Sci. Rep..

[15] Bossi P., Ascierto P.A., Basset-Seguin N., Dreno B., Dummer R., Hauschild A., Mohr P., Kaufmann R., Pellacani G., Puig S. (2023). Long-term strategies for management of advanced basal cell carcinoma with hedgehog inhibitors. Crit. Rev. Oncol. Hematol..

[16] Hanssen F., Garcia M.U., Folkersen L., Pedersen A.S., Lescai F., Jodoin S., Miller E., Seybold M., Wacker O., Smith N. (2024). Scalable and Efficient DNA Sequencing Analysis on Different Compute Infrastructures Aiding Variant Discovery. NAR Genom. Bioinform..

