# Supplementary material for: Resistance to SMO Inhibitors in Advanced Basal Cell Carcinoma: A Case Highlighting the Role of Molecular Tumor Profiling
Source: Int J Mol Sci. 2025 Dec 21;27(1):68. doi: 10.3390/ijms27010068 (PMC12785518; doi:10.3390/ijms27010068)
Supplement: Supplementary file 1 [file ijms-27-00068-s001.zip › ijms-4054700-supplementary.pdf]

DNA extraction and Next Generation Sequencing Analysis (NGS). Genomic DNA was extracted using the QIAamp DSP DNA FFPE Tissue Kit (Qiagen, Hilden, Germany) and quantified with a Qubit Fluorometer (Thermo Fisher Scientific) using the Qubit® dsDNA HS Assay Kit. A custom next-generation sequencing (NGS) panel (Illumina) was designed to target key genes involved in the Hh signaling pathway. For the custom panel, library preparation was performed using 15 ng of DNA with the Magnis NGS Prep System Agilent (Santa Clara, CA, USA), according to the manufacturer's guidelines. Sequencing was performed on the MiSeqDX platform (Illumina Inc, San Diego, CA, USA) using the MiSeq v2 Reagent Kit. For the NGS panel targeting 50 genes, the library was obtained using the AmpliSeq Library Plus for Illumina kit (Illumina). In this case, genomic DNA was analyzed using the 50-gene Ion AmpliSeq™ Cancer Hotspot Panel v2 (Thermo Fisher Scientific), according to the manufacturer's protocols. For data analysis, sequencing reads were processed with nf-core/sarek v3.4.3: alignment to GRCh37 was performed with BWA-MEM, duplicate marking applied, inline UMIs extracted with UMI-tools, and analysis restricted to the custom panel BED regions [1]. Somatic SNVs and indels were called with Mutect2 and Strelka2.

RT-PCR and gene expression analysis. Total RNA was extracted using the RNeasy FFPE kit for RNA extraction (Qiagen, Hilden, Germany) after a paraffin removing step. Complementary DNA (cDNA) was synthesized from 1 µg of total RNA using the FirstAid cDNA Synthesis Kit (Fermentas, Thermo Fisher Scientific, Waltham, MA, USA). All samples were tested in triplicate. Amplification of the β-actin transcript was included in all samples as an internal control. For each gene, the assessment of quality was performed by examining end-point PCR melt curves to ensure product specificity. Sequences of primers used were reported in Table S1.

[1]. Hanssen, F.; Garcia, M.U.; Folkersen, L.; Pedersen, A.S.; Lescai, F.; Jodoin, S.; Miller, E.; Seybold, M.; Wacker, O.; Smith, N. *et al.* Scalable and Efficient DNA Sequencing Analysis on Different Compute Infrastructures Aiding Variant Discovery. NAR Genom. Bioinform **2024**, *6*, lqae031.

Table S1

Primers for Real Time Quantitative Polymerase Chain Reaction

Target Gene	Foward Primer	Reverse primer
β-Actin NM_001101	5'-GACAGGATGCAGAAGGAGATTACT-3'	5'-TGATCCACATCTGCTGGAAGGT-3'
GLI1 NM_005269	5'-CAAAGTGGGAGGCACAAAC-3'	5'-TCCAGAATAGCCACAAAGTCC-3'
GLI2 NM_005270	5'-CAAGGCACCGCATCTGTGAT-3'	5'-GTCCTGGGTGGCAATCCTTG-3'
GLI3 NM_000168	5'-CCGCCGCAGGGCATTTT-3'	5'-CAGTGGTCGTGGAGCTGTG-3'
PTCH1 NM_001083602	5'-TGAAATCCAAGCCCAGCGTC-3'	5'-CAGTAGCCTTCCCCATAGCC-3'
PTCH2 NM_001166292	5'-AGATGTACAAGGAAAGCCCAGAG-3'	5'-GGCTAGATCTCCTCGGGCCTAA-3'
SUFU NM_001178133	5'-TGGACCCTGGTTACAACTCT-3'	5'-CCTCAGGGCAGAAAGACGTT-3'
BCL2 NM_000633	5'-GACAGAGGATCATGCTGTACTT-3'	5'-CCTTGGCATGAGATGCAGGA-3'
CCND1 NM_053056	5'-AATGACCCCGCACGATT-3'	5'GCACAGAGGGCAACGAAGG-3'
c-myc NM_002467	5'-TCAAGAGGCGAACACACAAC-3'	5'-GGCCTTTTCATTGTTTTCCA-3'
SOX2 NM_003106	5'-AGGATAAGTACACGCTGCCC-3'	5'-TAACTGTCCATGCGCTGGTT-3'
MTSS1 NM_014751	5'-CCTCCTCAAGTGAACAGGTGAT-3'	5'-TGCTGCAGACACTGGACTTT-3'
IGF-1 NM_000618	5'-ATCAGCAGTCTTCCAACCCA-3'	5'-TGGTGTGCATCTTCACCTTCA-3'
VEGF NM_001171630	5'-GTTGACCTTCCTCCATCC-3'	5'-TTCTCTGCCTCCACAATG-3'
IGFBP6 NM_002178	5'-GCCTGCTGTTGCAGAGGAGAA-3'	5'-GTTGGTCTCTGCGGTTCACA-3'
